# NREM parasomnias: a treatment approach based upon a retrospective case series of 512 patients

**DOI:** 10.1016/j.sleep.2018.03.021

**Published:** 2019-01

**Authors:** Panagis Drakatos, Lucy Marples, Rexford Muza, Sean Higgins, Nadia Gildeh, Raluca Macavei, Eptehal M. Dongol, Alexander Nesbitt, Ivana Rosenzweig, Elaine Lyons, Grainne d'Ancona, Joerg Steier, Adrian J. Williams, Brian D. Kent, Guy Leschziner

**Affiliations:** a Sleep Disorders Centre, Guy's Hospital, Great Maze Pond, London, SE1 9RT, United Kingdom; b Sleep and Brain Plasticity Centre, Department of Neuroimaging, IoPPN, King's College London, United Kingdom; c Faculty of Life Sciences and Medicine, King's College London, United Kingdom; d Department of Basic and Clinical Neurosciences, IoPPN, King's College London, United Kingdom

**Keywords:** NREM parasomnia, POD, Treatment, CBT, CPAP, Melatonin

## Abstract

**Background:**

Non-REM parasomnias are not uncommon conditions in the general population. Current treatment options are based on small case series and reports. In this study, we aimed to present the clinical experience from a large cohort of patients.

**Patients:**

Five hundred and twelve patients with Non-REM parasomnia or parasomnia overlap disorder (POD), who had undergone a video polysomnography and were exposed to treatment, were retrospectively identified. Treatment outcome was assessed based on patients’ reports, and treatment approach on a locally accepted hierarchy of interventions.

**Results:**

Forty percent of patients were diagnosed with sleepwalking, 23.8% with mixed-phenotype and 10% with POD. Ultimately, 97.2% reported adequate control of their symptoms. Moreover, 60.1% were treated with pharmacotherapy and 32.0% without, consistent across all phenotypes (p = 0.09). Benzodiazepines were the most common drugs prescribed (47.1%, p < 0.05). In the end, 37.7% of our patients were receiving a benzodiazepine as part of their successful treatment, 11.7% an antidepressant, 9.2% a z-drug, and 10.7% melatonin. Finally, 13.2%, 12.1%, and 5.8% of our patients reported good control of their symptoms with sleep hygiene, management of sleep-disordered breathing, and psychological interventions (cognitive behavioral therapy [CBT] or mindfulness-based stress reduction [MBSR]), as monotherapy, respectively.

**Conclusion:**

The treatment approach to effective treatment of the patients with Non-REM parasomnias or POD offering first sleep hygiene advice, next treatment of concurrent sleep disorders and management of other priming factors like stress and anxiety, and lastly pharmacotherapy for Non-REM parasomnia is supported by our results. Non pharmacological interventions were effective in one third of our patients, and CBT/MBSR and melatonin appeared promising new treatments.

## Highlights

• This study supports a standardised treatment approach in NREM-parasomnias.
• Pharmacotherapy alone effectively treated two thirds of the patients.
• One third of the patients were effectively treated without pharmacotherapy.
• Treatment of sleep apnea and periodic limb movements in sleep is recommended.
• Melatonin and psychological therapies are promising treatment options.

## 1 Introduction

Non rapid eye movement (NREM) parasomnias are abnormal behaviors arising primarily but not exclusively during non-REM stage three (N3) sleep. Phenotypes include sleepwalking, sleep terrors, confusional arousals, sexsomnia, and sleep-related eating disorder (SRED). Patients may also present with concurrent REM parasomnia, in a subtype of REM behaviour disorder (RBD) termed parasomnia overlap disorder (POD) [1], [2], [3].

Whilst the exact mechanism underlying NREM parasomnias is unknown, a number of predisposing, priming, and precipitating factors have been identified [4]. The mainstays of management of the NREM parasomnias are modulation of these factors and ensuring safety. Predisposing factors are thought to be primarily genetic, with many patients reporting a family history, and more recently genetic covariation between different phenotypes has been confirmed [5], [6], [7], [8]. Thus, these factors are problematic to address. Priming factors typically increase the proportion and depth of N3 sleep or make arousal from it more difficult. It is postulated that these priming factors create a favourable environment for internal or external stimuli (precipitating factors) to trigger a partial arousal of the brain, manifesting as complex behaviors accounting for NREM parasomnia phenotypes. Potent priming factors are thought to include sleep deprivation, medications such as hypnotic drugs, and stress [4]. Precipitating factors include noise, touch from a bed partner, sleep disordered breathing (SDB) and periodic limb movements in sleep (PLMS) [4], [9].

Standard management strategies include a safety plan, reassurance and general measures to improve sleep hygiene. Patients should be advised on avoidance of sleep deprivation, caffeine and alcohol consumption (especially close to bed-time), moreover, medications should be reviewed for known interactions [4], [10], [11].

Treating co-morbid sleep disorders offers an alternative strategy to improving NREM parasomnias. Obstructive sleep apnea (OSA), even of mild severity, or upper airway resistance syndrome, can precipitate these nocturnal phenomena, and continuous positive airway pressure (CPAP) treatment, mandibular advancement devices or upper airway tissue reduction surgeries have proven to be effective especially in patients with sleep walking [12]. Restless legs syndrome (RLS) and PLMS have been particularly associated with SRED, and dopaminergic agents and alpha-2-delta ligands are considered first-line treatments [13], [14]. Insomnia can also be effectively treated with cognitive behavioral therapy for insomnia(CBTi) [15], [16].

Situational stress frequently triggers NREM parasomnias, and cognitive behavioral therapy for stress and anxiety (CBTs-a) can effectively alleviate this factor [17]. Mindfulness-Based Stress Reduction (MBSR) has been in the spotlight over the past few years, and there is evidence that it can improve sleep, especially when it is practiced regularly [18], [19]. Traditionally, hypnotherapy has been used to treat patients with NREM parasomnias, especially with sleep walking [20]. Anticipatory awakening before the time that NREM parasomnia events are expected to occur has also been utilised therapeutically [21].

In severe cases however, or those refractory to initial management, pharmacotherapy is valuable. Although presently the evidence basis for these approaches consists primarily of a limited number of case reports and case series, with small numbers of patients and often contradictory results [14], [22]. Commonly prescribed medications include benzodiazepines or benzodiazepine receptor agonists, and antidepressants. A major issue is that these classes of medications can exacerbate NREM parasomnias, worsen precipitating factors for parasomnias (such as SDB and PLMS), and can increase daytime somnolence [4], [23], [24], [25]. Clonazepam is commonly the first-line pharmacotherapy both for NREM parasomnias and RBD [14], [22], [26], [27], [28]. Antidepressants, including the selective serotonin reuptake inhibitors (SSRIs), tricyclics (TCAs) and the related drug trazodone may be of use in treating NREM parasomnias [4], [24]. Additionally, melatonin can effectively re-align a delayed circadian rhythm, treat insomnia and RBD, and potentially improve sleep terrors and sleep walking based on a handful of case reports in children [16], [28], [29], [30].

The aim of this study was to assess the effectiveness of various treatments prescribed to a large unselected cohort of patients with a diagnosis of NREM parasomnia with or without coexisting RBD. In the absence of large prospective studies, we intended to expand the evidence base of pharmacological and non-pharmacological interventions in these conditions.

## 2 Material and methods

### 2.1 Patient selection

Using an internal sleep laboratory database, we retrospectively identified adult patients with a likely diagnosis of NREM parasomnia, irrespective of other concomitant sleep disorders, following a consultation with an experienced sleep physician at the Sleep Disorders Centre, Guy's and St Thomas' Hospitals, London, UK, over a period of 7.5 years (between June 2008 and December 2015). Appropriate approval from the institutional review board on human research was obtained (project number 8126).

**Fig. 1:**
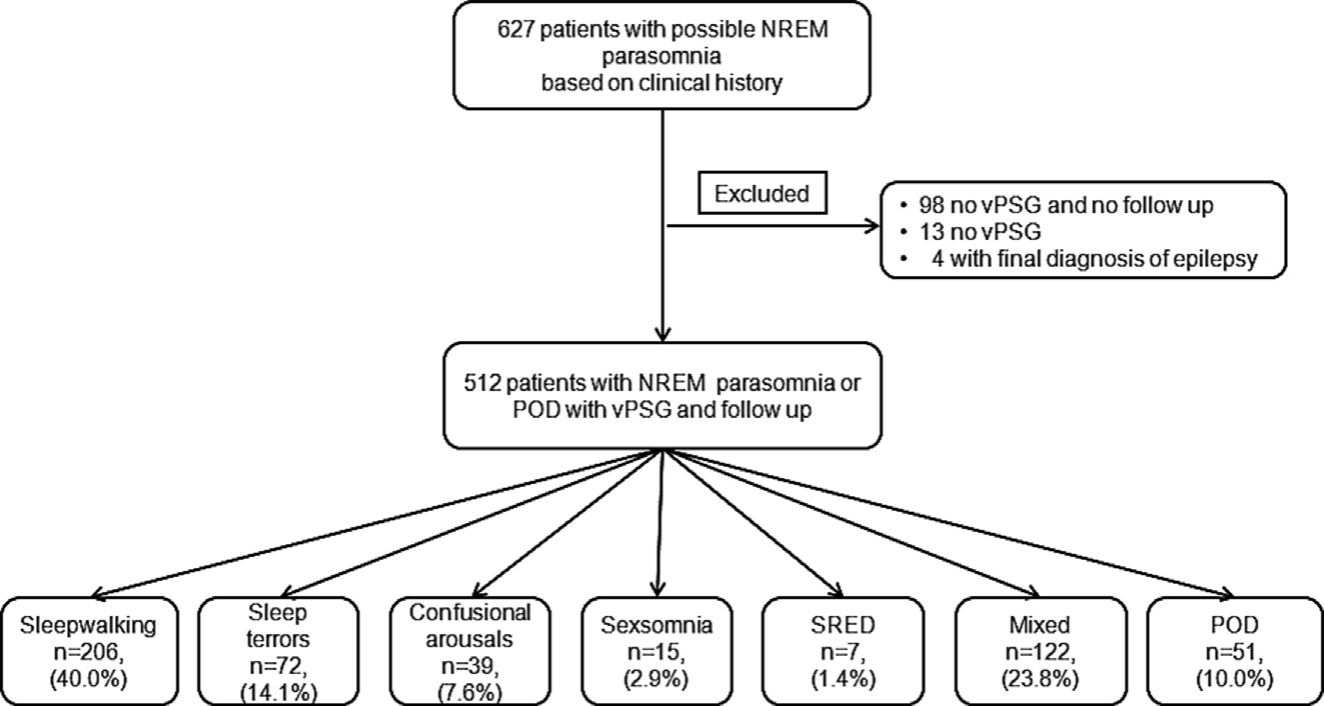
Flow diagram of the studied cohort. Percentages indicate the prevalence of each diagnostic group in our cohort of 512 patients. NREM, non-REM; POD, parasomnia overlap disorder; vPSG, video polysomnography; SRED, sleep related eating disorder; Mixed, mixed phenotypes of NREM parasomnia; n, number.

All cases were retrospectively reviewed, and patients with a final diagnosis of NREM parasomnia or POD based on International Classification of Sleep Disorders third edition (ICSD-3) criteria who had undergone a video polysomnography (vPSG), were included in the analysis and divided into six major categories (Fig. 1) [1]. Patients with coexisting phenotypes of NREM parasomnias were grouped as mixed. None of the patients had received a prior diagnosis or treatment for NREM parasomnia, POD or other sleep disorders, except from patients with insomnia diagnosed in primary care and exposed to hypnotic agents, in order to explore the significance of the latter as priming factors for NREM parasomnia. Patients without follow up (FU) or lost to FU were excluded from the analysis. Demographics, medical history and medications of the cohort were derived from medical records. A standardised diagnostic approach was shared between clinicians with the intention to order vPSG for all patients.

### 2.2 Treatment

All patients received general safety recommendations and sleep hygiene advice prior to initiation of any other treatment. A sleep hygiene leaflet which included but was not limited to measures on or around bedtime habits, keeping a regular sleep/wake pattern and adequate sleep opportunity was offered to all the patients. Sleep hygiene effectiveness was assessed as an overall outcome and individual measures were not assessed separately. Further treatment was guided by clinically or polysomnographically identified concurrent sleep disorders and attention was paid on managing stress and anxiety with or without pharmacotherapy. Patients who had been refractory to any intervention up to this point, or without obvious priming and precipitating factors, were initiated on or trialed on different classes of medications, without a formal consensus on the sequence or the type of drug used. MBSR and melatonin were only utilised for the last two years of the study period. Treatment success was based on patients' subjective reports of either total resolution or satisfactory control of their parasomnia symptoms, and without experiencing drug-related side effects that would have limited their usage. Medical records were searched for priming factors associated with NREM parasomnia, without a pre-established list, including sleep deprivation, stress, insomnia, hyperthyroidism, migraines, history of head injury, encephalitis and stroke, and drugs including lithium, phenothiazines, anticholinergic agents and hypnotic drugs. Sleep deprivation was captured through sleep diaries or/and home actigraphy (7-/14-days), and stress was self-reported. The diagnosis of insomnia was made based on the existing ICSD criteria at the time of the diagnosis.

The following precipitating factors, including external stimuli such as noise, light and bed partner's movements, and internal stimuli including SDB and PLMS, were extracted from patients' records without a pre-established list, and vPSG respectively.

### 2.3 Sleep study methodology

Attended inpatient vPSG was performed using standard 10–20 EEG montage, with sleep stages scored according to the standard criteria of the American Academy of Sleep Medicine (AASM). Continuous recordings included electrooculography, electrocardiography, submental and leg electromyography, pulse oximetry, nasal pressure cannulae, and respiratory inductance plethysmography, with chest and abdominal belts [31]. Time-synchronized video recordings were also performed, while audio was captured by a multidirectional microphone placed above the patient. In patients requiring multiple sleep latency testing for diagnostic purposes of concomitant sleep disorders, standard guidelines were applied [32].

Apneas and hypopneas, periodic limb movements in sleep (PLMS), and REM without atonia (RWA), were scored following standard AASM definitions to determine the apnea–hypopnea index (AHI) and PLM-index (PLMI), using as cut-offs the five events/h and 15 events/h for the diagnosis of obstructive sleep apnea (OSA) and PLMS respectively [31].

All cases of concomitant diagnosis of hypersomnia of central origin were retrospectively reviewed and diagnosis was made *de novo* according to ICSD-3 criteria [1]. These patients had presented at the time of first consultation both with NREM parasomnia and hypersomnia of central origin features and they were treatment naive.

### 2.4 Statistical analysis

Data are reported as mean ± standard deviation (SD) if not otherwise indicated. Following testing for normality, comparisons between the six groups were performed using the Kruskal–Wallis test, with Dunn's Multiple Comparison Test when needed. A Chi Square test was used for correlations between variables. A value of p < 0.05 was considered to be statistically significant. IBM SPSS Statistics V24.0 (SPSS, Chicago, IL/USA) was used for all statistical analysis.

## 3 Results

Based on clinical history, 627 patients with a possible diagnosis of NREM parasomnia were identified. Five hundred and twelve patients [265 males (51.8%)] with age ranging from 19 to 88 years old and with a mean of 39.3 ± 12.1 years, had undergone a vPSG and had their diagnosis secured, and were exposed to treatment with adequate follow up time of at least six months [median 8.0 months (interquartile range 7.0–11.0) of follow up time (FU)] (Fig. 1). 40.0% (206/512) were diagnosed with sleepwalking alone, and 23.8% (122/512) had a mixed phenotype (Fig. 1). POD patients were the oldest among the groups (p = 0.023). A clear male predominance was observed in the sexsomnia group (87.7%, p = 0.017) (Table 1). Twenty-two percent (113/512) were diagnosed with OSA (AHI: 18.6 ± 15.5 events/h) and 11.1% (57/512) with PLMS (PLMI: 33.2 ± 23.6 events/h), and there was no difference between studied groups (p > 0.05, Table 1); patients in the SRED cohort did not have a higher prevalence of PLMS.

**Table 1:** Demographics and concomitant sleep disorders of the studied groups.

	Diagnosis							P value
	Sleepwalking n = 206	Sleep terrors n = 72	Confusional arousals, n = 39	Sexsomnia n = 15	SRED n = 7	Mixed n = 122	POD n = 51	
**Demographics**								
Age (y)	38.5 ± 11.3	38.9 ± 14.3	42.9 ± 14.3	36.9 ± 8.0	43.8 ± 18.2	37.2 ± 10.6	45.1 ± 14.9	0.023^a^
Gender (m, %)	52.4	40.3	53.8	86.7	28.6	49.2	62.7	0.017^b^
BMI (Kg/m^2^)	27.3 ± 5.1	25.7 ± 4.6	29.1 ± 7.2	25.9 ± 5.0	26.2 ± 5.3	27.4 ± 4.7	27.2 ± 4.0	0.203
ESS	9.6 ± 5.8	9.1 ± 4.4	11.5 ± 6.6	9.9 ± 6.5	8.5 ± 6.0	8.7 ± 5.2	10.5 ± 4.8	0.201
**Concomitant sleep disorders**								
OSA, n (%)	45 (21.8)	12 (16.7)	13 (33.3)	7 (46.7)	2 (28.6)	21 (17.2)	13 (25.5)	0.072
PLMS, n (%)	20 (9.7)	4 (5.6)	6 (15.4)	4 (26.7)	1 (14.3)	12 (9.8)	10 (19.6)	0.087
NT1/NT2/IH, n	1/1/5	0/0/1	0/2/1	0 (0)	0 (0)	0 (0)	0 (0)	0.063

SRED, sleep related eating disorder; Mixed, mixed phenotypes of NREM parasomnia; NREM, non-REM; POD, parasomnia overlap disorder; ESS, Epworth sleepiness scale; OSA, obstructive sleep apnea; PLMS, periodic limb movements during sleep; NT1, narcolepsy type 1; NT2, narcolepsy type 2; IH, idiopathic hypersomnia; y, years; m, male; n, number.

a Mixed versus POD (p = 0.027) using Independent Samples Kruskal–Wallis Test with Dunn's Multiple Comparison Test.

b Chi Square with Cramer's V product.

Furthermore, 97.2% (498/512) of the patients reported that their parasomnia manifestations were at least under an acceptable level of control for them individually or they have totally resolved; and 32.0% (164/512) did not require pharmacotherapy, using either sleep hygiene, CPAP/MAD, CBT/MBSR, acupuncture or by discontinuing drugs deemed responsible for triggering NREM parasomnia. Pharmacotherapy alone, as monotherapy or combination of drugs, targeting the NREM parasomnia or concomitant sleep disorders like PLMS, treated the 60.1% of the cohort, and that was independent of the diagnosis (p = 0.09) (Table 2).

**Table 2:** Type of successful treatment used per diagnosis.

Treatment options	Diagnosis							Total n, (%)
	Sleepwalking n, (%)	Sleep terrors n, (%)	Confusional arousals, n, (%)	Sexomnia n, (%)	SRED n, (%)	Mixed n, (%)	POD n, (%)	
Pharmacotherapy^a^	122 (59.2)	42 (58.3)	16 (41.0)	9 (60.0)	2 (28.6)	82 (67.2)	35 (68.6)	308 (60.1)
Sleep hygiene advice	37 (17.9)	11 (15.2)	10 (25.6)	0	0	8 (6.5)	2 (3.9)	68 (13.2)
CPAP/MAD	22 (10.6)	6 (8.3)	9 (23.1)	3 (20.0)	1 (14.3)	13 (10.6)	8 (15.6)	62 (12.1)
CBTi/CBTs-a/MBSR	12 (5.8)	6 (8.3)	2 (5.1)	1 (6.6)	1 (14.3)	8 (6.5)	0	30 (5.8)
Combination of treatments^b^	10 (4.8)	2 (2.8)	2 (5.1)	0	3 (42.9)	7 (5.7)	2 (3.9)	26 (5.0)
Discontinuation of medication	1 (0.5)	1 (1.4)	0	0	0	0	1 (1.9)	3 (0.6)
Acupuncture	0	1 (1.4)	0	0	0	0	0	1 (0.2)
Failed treatment	2 (0.9)	3 (4.1)	0	2 (13.3)	0	4 (3.2)	3 (5.8)	14 (2.7)
Total	206 (100)	72 (100)	39 (100)	15 (100)	7 (100)	122 (100)	51 (100)	512 (100)

SRED, sleep related eating disorder; Mixed, mixed phenotypes of NREM parasomnia; NREM, non-REM; POD, parasomnia overlap disorder; CPAP, continuous positive airway pressure; MAD, mandibular advancement device; CBTi, cognitive behavioral treatment for insomnia; CBTs-a, cognitive behavioral treatment for stress and anxiety; MBSR, mindfulness-based stress reduction program.

a Monotherapy or combination of drugs.

b Combination of pharmacological treatment and not on top of sleep hygiene advice.

The type of successful treatment differed between phenotypes (p < 0.0001, Table 2). The majority of the patients for all groups except SRED, were treated with pharmacotherapy alone, after sleep hygiene advice. Notably, 48.7% of the patients with confusional arousals were treated with CPAP/MAD or sleep hygiene advice alone, while three out seven patients with SRED required combination of treatments and one out of seven reported successful control of the symptoms after attending a course for cognitive behavioral treatment for insomnia (Table 2).

### 3.1 Sleep hygiene

**Fig. 2:**
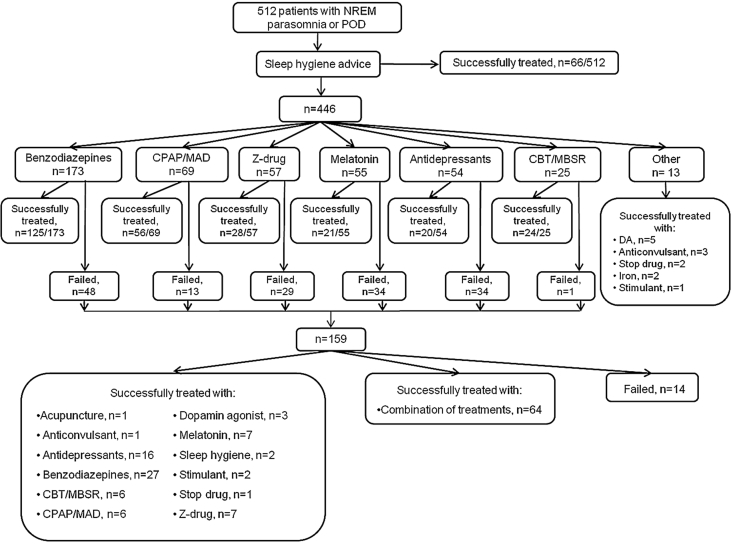
Treatment approach and outcome for 512 patients with NREM parasomnia or POD. NREM, non-REM; POD, parasomnia overlap disorder; CPAP, continuous positive airway pressure; MAD, mandibular advancement device; CBT, cognitive behavioral treatment; MBSR, mindfulness-based stress reduction program; DA, dopamine agonist; Anticonvulsant, pregabalin or gabapentin; Stimulant, modafinil; Combination of treatments, combination of more than one treatment options.

Moreover, 13.2% of our patients, primarily those with confusional arousals, sleepwalking and sleep terrors (p = 0.003) had to implement only sleep hygiene in their daily life in order to achieve satisfactory control of their symptomatology (Table 2, Table 3 and Fig. 2).

**Table 3:** Therapeutic options and outcome.

Pharmacotherapy	Treatment outcome and information						
	Exposed n, (%)	Success Rate n, (%)	P value^b^ per diagnosis	As a standalone treatment n, (%)	Treated with n, (%)	Commonest drug/treatment (%)	Dose mg ± SD
Benzodiazepines	241/512 (47.1)	193/241 (80)	0.933	152/193 (78.8)	193/512 (37.7)	Clonazepam (95.8)	0.63 ± 0.64
Z-drugs	81/512 (15.8)	47/81 (58)	0.728	35/47 (74.5)	47/512 (9.2)	Zopiclone (100)	4.14 ± 1.16
Antidepressants	97/512 (18.9)	60/97 (61.8)	0.551	36/60 (60.0)	60/512 (11.7)	Fluoxetine (31.6)	13.3 ± 4.9
Melatonin, PR	71/512 (13.9)	55/71 (77.4)	0.066	28/55 (50.9)	55/512 (10.7)	N/A	2.4 ± 1.0
Dopamine agonist	10/512 (1.9)	8/10 (80)	0.040	8/8 (100)	8/512 (1.5)	Ropinirole (62.5)	1.4 ± 0.5
Pregabalin/Gabapentin^a^	5/512 (0.9)	4/5 (80)	0.659	4/4 (100)	4/512 (0.7)	Pregabalin (50)	300 ± 0
CPAP/MAD	92/512 (17.9)	79/92 (85.8)	0.105	62/79 (78.4)	79/512 (15.4)	CPAP (72.5)	N/A
CBTi/CBTs-a/MBSR	40/512 (7.8)	32/40 (0.8)	0.412	30/32 (0.93)	30/512 (5.8)	CBTi (42.5)	N/A
Sleep hygiene advice	512/512 (100)	N/A	<0.001	68/512 (13.2)	N/A	N/A	N/A

SD, standard deviation; n, number; SSRIs, serotonin selective reuptake inhibitors; SARI, serotonin antagonist and reuptake inhibitor; TCAs, tricyclic antidepressants; PR, prolonged-release; CPAP, continuous positive airway pressure treatment; MAD, mandibular advancement device; CBTi, cognitive behavioral treatment for insomnia; CBTs-a, cognitive behavioral treatment for stress and anxiety; MBSR, Mindfulness-based stress reduction program; N/A, not applicable.

a These medications were prescribed for restless leg syndrome/periodic limb movement disorder.

b P value reflects on success rate comparison between different diagnoses and was calculated using Chi Square test with Cramer's V product and Fischer's exact test as required.

### 3.2 Pharmacotherapy

Four hundred and forty-six out of 512 patients required further intervention following the implementation of sleep hygiene, and pharmacotherapy was utilized next in 350 of them (78.4%). Benzodiazepines were first drugs most commonly used [173/350 (49.4%)] with the best success rate at 72.2% (p < 0.001) compared to z-drugs, antidepressants and melatonin (49.1%, 37% and 38.2%, respectively) (Fig. 2). Overall, almost half of our patients were exposed to benzodiazepines, primarily clonazepam, and 80% had a good response irrespective of the diagnosis (p = 0.933). In addition, 37.7% of our patients received a benzodiazepine as part of their successful treatment combination (Table 3), and 29.7% (152/512) of the patients were treated with benzodiazepine alone (Fig. 2).

Zopiclone, was constituted as a successful treatment either in part or in whole for 9.2% of the patients irrespective of their diagnosis (p = 0.728). Of the 15.8% who were exposed to the drug two thirds of them responded well, with the majority receiving it as monotherapy (Table 3). Following sleep hygiene advice, 12.7% of patients received zopiclone and half of them (49.1%) had their symptoms successfully controlled (Fig. 2).

Antidepressants were prescribed in 18.9% of our patients and 11.7% were receiving an antidepressant as part of their successful treatment, irrespective of their diagnosis (p = 0.551). Fluoxetine was the most common antidepressant prescribed [31.7% (19/60), Table 3] followed by citalopram (20.0%), mirtazapine and trimipramine (8.3%) amitriptyline (6.7%), paroxetine, sertraline, clomipramine and trazodone (5.0%) imipramine (3.3%), and venlafaxine (1.6%). As their first intervention, 21.1% of our patients received an antidepressant as following sleep hygiene advice and a third of them (37%) responded well (Fig. 2).

Fifty-five out of 512 patients (10.7%) were treated with melatonin which was provided in a sustained release preparation. The majority consisted of patients with POD (32.7%), sleepwalking (29.1%), and mixed phenotype (21.8%) (p < 0.001, Table 4). Half of the patients that were successfully treated with melatonin, were receiving it as monotherapy (Table 3), and these were primarily patients with POD, sleepwalking and mixed phenotype (p < 0.001, Table 4). Similar to antidepressants and z-drugs, a 12.1% of our patients that failed sleep hygiene were prescribed melatonin and 38.2% reported good control of their symptoms (Fig. 2).

**Table 4:** Patients successfully treated with melatonin.

Melatonin MR	Diagnosis							P value
	Sleepwalking n, (%)	Sleep terrors n, (%)	Confusional arousals n, (%)	Sexsomnia n, (%)	SRED n, (%)	Mixed n, (%)	POD n, (%)	
Treated with (n = 55)	16 (29.1)	3 (5.4)	4 (7.3)	0	2 (3.6)	12 (21.8)	18 (32.7)	<0.001
As a standalone treatment (n = 28)	6 (21.4)	2 (7.2)	4 (14.3)	0	0	6 (21.4)	10 (35.7)	<0.001

MR: modified release; SRED, sleep related eating disorder; Mixed, mixed phenotypes of NREM parasomnia; POD, parasomnia overlap disorder.

*; P value was calculated using Chi Square with Fischer's exact test.

Fifty-seven out of 512 (11.1%) of the patients were diagnosed with PLMS, and 26.3% of them (15/57) received treatment targeting PLMS (eight received dopamine agonist, four pregabalin, two had iron supplementation and one had SSRI-related PLMS which resolved after discontinuation of the drug) with subsequent good control of the nocturnal abnormal manifestations (Table 3 and Fig. 2). Another 29.8% (17/57) were treated with benzodiazepine alone, and 15.8% (9/57) with zopiclone or a combination of sedative drugs. In 17.5% of the patients with PLMS, CPAP/MAD treatment was applied for concomitant OSA offering good control of the parasomnia symptoms, and in three more cases benzodiazepine had to be added on top of CPAP/MAD. One patient with PLMS did not receive any treatment except from sleep hygiene advice and general recommendations, while two patients failed treatment with benzodiazepine and zopiclone. PLMI did not differ significantly between those received PLMS-targeted treatment and those that did not (p = 0.508).

We identified three cases of drug-related NREM parasomnias; one with SSRI induced PLMS and another two with sleepwalking, and sleep terrors receiving zaleplon for insomnia. In all three cases the NREM parasomnia resolved after the discontinuation of the drugs.

### 3.3 CPAP/MAD

One hundred and thirteen out of 512 (22.0%) of patients were diagnosed with OSA and 81.4% of those received either CPAP if at least moderate OSA was confirmed, or a custom made MAD for milder conditions. The rest of the patients declined or could not tolerate any of the two treatments (n = 21) and had a significantly lower AHI (21.6 ± 16.3 vs 8.3 ± 2.2 events/h, p < 0.001). Of those, 85.7% of patients receiving any of the two treatments had satisfactory control both of OSA and their parasomnia events. Moreover, in 78.4% of these cases no other treatment was required (CPAP, n = 45; MAD, n = 17). The success rate was similar between studied groups (p = 0.105). AHI and compliance did not differ significantly between those reporting good response to CPAP/MAD and those that did not (p = 0.669 and p = 0.178) (Table 3). Seventy-five percent of the patients that were exposed to CPAP/MAD had this treatment applied as first intervention following sleep hygiene advice, and the majority (81.1%) of them required no further treatment (Fig. 2).

### 3.4 CBTi, CBTs-a, MBSR

Forty patients were referred to attend a CBT or MBSR, with a success rate of 80% (32/40) which did not differ between groups (p = 0.412). Of those that were successfully treated, 13 had CBTi, 10 CBTs-a, and seven MBSR not requiring any additional treatment. The selection of the patients was based on clinician's preference when increased levels of stress and anxiety were identified, or when insomnia was diagnosed and the sleep study had excluded other concomitant sleep disorders. Twenty-five out of 40 that had a CBT or MBSR, attended the course following sleep hygiene advice and prior to any other treatment, and all but one patient reported successful control of their symptoms (Fig. 2).

## 4 Discussion

This study reports the largest cohort of patients diagnosed and treated for NREM parasomnias currently described in the literature, covering the full spectrum of the condition, including those with POD. The treatment approach started with sleep hygiene advice and a safety plan, followed by treatment of concurrent sleep disorders and of other priming and precipitating factors, and when required by NREM parasomnia or POD-targeted pharmacotherapy, yielding a 97.2% subjective satisfaction of symptom control for our patients. Pharmacotherapy alone, as monotherapy or a combination of drugs, remained a pivotal component of successful treatment for our patients (60.1%), but it should be highlighted that one third of the patients did not require any pharmacotherapy. This study strengthens the existing data on effectiveness of different types of drugs and interventions, and also introduces melatonin as a treatment of NREM parasomnia in adults, and CBT/MBSR in selected cases with promising results. The type of successful treatment differed with diagnosis (p < 0.0001) giving rise to more tailored and phenotype-guided treatment options in future clinical practice.

Reassurance, safety measures and sleep hygiene advice should be the first line of treatment for patients with NREM parasomnia. In this study, 12.9% of our cohort did not require any further interventions, and ultimately 25.6% of the patients with confusional arousals, 17.9% with sleepwalking and 15.2% with sleep terrors, were treated successfully using this approach. Reassurance should include explaining to patients that the nocturnal manifestations of NREM parasomnia are not part of a psychopathology, while plans to avoid injuries are also discussed, like locked windows and bedroom doors or use of bedroom door alarms, removal of bedside objects that could cause injuries, and advice to avoid forced awakening of the patient during a NREM parasomnia event [14]. Sleep deprivation is a common trigger of NREM parasomnias; and sleep hygiene leaflets are easily accessible over the internet and should ideally be offered at the stage of primary care assessment. In cases where NREM parasomnia overlaps with RBD or RWA, information should be offered that the latter two can often predate the development of Parkinson's disease and other disorders of synuclein pathology. Assessment of these patients for early signs of these conditions like constipation and olfactory deficiencies is recommended [33], [34].

One hundred and sixty out of 512 (31.2%) of the patients were diagnosed with at least one concomitant sleep pathology (OSA, PLMS and hypersomnia of central origin) following vPSG and MSLT when required. Ninety-one out of 512 (17.8%) received treatment targeting their concurrent sleep pathologies, and reported satisfactory control of the abnormal nocturnal manifestations, irrespective of their NREM-parasomnia phenotype and the severity of OSA or PLMS; our findings are in line with existing reports. Moreover, this is the first time that treatment of OSA and PLMS in NREM parasomnias is supported on a large scale, irrespective of the NREM-parasomnia phenotype [12], [13], [14]. These results would support the recommendation included in the ICSD3 of performing vPSG in complicated NREM parasomnia cases where another sleep diagnosis, like OSA and PLMS may coexist [1].

Hypnotherapy aims to induce physical relaxation, and based on current literature has been utilised successfully in managing a few patients with NREM parasomnias and rarely with POD [35], [36]. CBTs-a and CBTi are based on psychosocial interventions and they focus on reducing stress and anxiety levels, and resolve insomnia, respectively, which are all well-known precipitating factors for NREM parasomnias. Along the same lines, MBSR which helps individuals self-manage and reframe worrisome and intrusive thoughts, aims at stress reduction and has been a promising treatment for insomnia [18], [19]. Since hypnotherapy was not easily accessible to our patients, 40 patients without OSA, PLMS or central hypersomnia, and with the aforementioned priming factors present, were selected to attend a type of CBT or MBSR as felt to be clinically appropriate. Thirty two responded well and 30 did not require any additional treatment. Among them there were two pregnant women, and these non-pharmaceutical interventions would be an ideal option for this sub-group of patients. While we acknowledge the selection bias and the limitation of the study design to compare treatments, our results provide enough clinical support that these interventions could be viable treatment options and further studies will be required to give credence to that.

In line with existing data, clonazepam was the drug most commonly used (37.7%, p < 0.001), and pharmacotherapy alone was the primary source of successful treatment for our patients (60.1%) [26], [37]. The success rate of clonazepam (72.2%) whenever used as a first line drug, and compared to the rest of the drugs (p < 0.001), would support our clinical experience that this should be a first line option especially when patients or bed-partners have sustained or are likely to sustain injuries, and after other sleep pathologies have been excluded or treated, and risk of fall or drug abuse has been taken into consideration [26], [38].

Hypnotic drugs, especially zolpidem and zaleplon, have been linked to SRED and other NREM parasomnias [4], [11]. Notably, zopiclone was offered to 81 of our patients and 47 (9.2% of the cohort) were successfully treated, with the addition of other treatments when needed. None of the patients that failed to respond to zopiclone reported any noticeable increase in the frequency of nocturnal events. Therefore, zopiclone could be considered a treatment option for these patients, especially when patients require pharmacotherapy and suffer from concomitant insomnia too. Α difference in pharmacodynamics between z-drugs could offer a possible explanation for the discrepancies mentioned above. This hypothesis could be based on the recently described paradoxical effect of zolpidem in patients with OSA where muscle activity was increased during airway narrowing compared to zopiclone and placebo, while the arousal threshold was increased similarly for both of the z-drugs [39].

Eleven antidepressants, including SSRIs and TCAs, were used as part of the successful treatment of 11.7% of our patients. The most commonly used were fluoxetine, citalopram and mirtazapine [14], [22]. None of the pharmaceutical categories above (benzodiazepines, z-drugs, antidepressants) showed different effectiveness per diagnosis (p > 0.05, Table 3).

Prior to introducing melatonin to our patients with NREM parasomnia, we observed cases of POD where patients receiving this drug were reporting adequate control both of RBD and NREM parasomnia events. In this study, 10.7% of our cohort were treated successfully with melatonin (Table 3, Fig. 2). The majority of these patients were suffering from POD (32.7%, p < 0.001), but notably there were another 29.1% suffering from sleepwalking and 21.8% with mixed phenotype (Table 4). We hypothesize that sleep consolidation, treatment of sleep deprivation (4/55 of patients treated with melatonin) or of concomitant insomnia (3/55), are potential effects by which melatonin is managing to control NREM parasomnia [30], [40]. Considering the good safety profile and these promising results, especially when compared to z-drugs or antidepressants, melatonin is proposed as one of the first line pharmacotherapy options in patients with NREM parasomnias or POD, however, future studies will be required to support this statement.

In summary, these data provide supportive evidence for standard therapies proposed for NREM parasomnias, but also lend support for the use of melatonin, zopiclone and psychological therapies such as CBT and MBSR. Furthermore, these data also support the tackling of sleep comorbidities such as SDB and RLS/PLMS before utilizing specific pharmacotherapies directed at NREM parasomnias.

### 4.1 Limitations

There are several limitations to our study. First, the study design was retrospective and treatment success was based on patients' reports and their ability to recollect past events considering the retrograde amnesia usually associated with NREM parasomnia, which may explain the observed high success rate. Nonetheless, the essence of the treatment aim in patients with parasomnias in clinical practice is safety and addressing patients' symptoms adequately as per their criteria. Validated questionnaires on parasomnia severity, such as the Munich Parasomnia Screening (MUPS) questionnaire, would be recommended for future prospective studies adding objective data to our results [41]. The utilization of a standardised list of predisposing, priming and precipitating factors would also be helpful in future studies.

In the context of a retrospective study, while CPAP compliance was confirmed, CBT and MBSR attendances and medication compliance were not, and this may have had an impact on our results. We also acknowledge that patients that received CBT or MBSR were subject to selection bias, and so these results should not be generalised, although blanket psychological therapies for NREM parasomnias could be the focus of future studies. Finally, this study provides information on drugs' effectiveness based on clinical practice and not on direct comparison between different treatment elements.

## 5 Conclusion

Based on a high success rate our findings would support a standardised treatment approach for patients with NREM parasomnia or POD. Treatment of concomitant sleep disorders and, in particular, of OSA even in mild forms should be a priority. Clonazepam was the most commonly used and effective drug irrespective of diagnosis, and antidepressants and zopiclone remain viable options. Results from melatonin administration were promising and its safety profile may promote this medication as first line pharmacotherapy option in the future. CBT and MBSR can be considered prior to or as an alternative to pharmacotherapy in selected patients.

## Authors' contributorship statement

AW, GL, PD, and BK: concept and study design; LM, EM and PD: Data acquisition and analysis; All authors contributed substantially to interpreting the results, drafting the article and revising it critically for intellectual content and gave final approval to the submitted manuscript.
