# Corrigendum to “NREM parasomnias: a treatment approach based upon a retrospective case series of 512 patients” [Sleep Med 53 (2019) 181–188]

**DOI:** 10.1016/j.sleep.2019.07.001

**Published:** 2020-01

**Authors:** Panagis Drakatos, Lucy Marples, Rexford Muza, Sean Higgins, Nadia Gildeh, Raluca Macavei, Eptehal M. Dongol, Alexander Nesbitt, Ivana Rosenzweig, Elaine Lyons, Grainne d'Ancona, Joerg Steier, Adrian J. Williams, Brian D. Kent, Guy Leschziner

**Affiliations:** a Sleep Disorders Centre, Guy's Hospital, Great Maze Pond, London, SE1 9RT, United Kingdom; b Sleep and Brain Plasticity Centre, Department of Neuroimaging, IoPPN, King's College London, United Kingdom; c Faculty of Life Sciences and Medicine, King's College London, United Kingdom; d Department of Basic and Clinical Neurosciences, IoPPN, King's College London, United Kingdom

The authors regret that the information Ivana Rosenzweig was supported by Wellcome Trust Grant [103952/Z/14/Z] was not included in the published article. The authors would like to apologise for any inconvenience caused.

